# Epigallocatechin-3-Gallate Protects HUVECs from PM_2.5_-Induced Oxidative Stress Injury by Activating Critical Antioxidant Pathways

**DOI:** 10.3390/molecules20046626

**Published:** 2015-04-14

**Authors:** Guang-Zhao Yang, Zhao-Jun Wang, Feng Bai, Xiao-Jiang Qin, Jing Cao, Ji-Yuan Lv, Ming-Sheng Zhang

**Affiliations:** 1 The Fist Clinical Hospital, Shanxi Medical University, 56 Xinjiannanlu, Taiyuan, Shanxi 030001, China; E-Mails: ygzloveqq@126.com (G.-Z.Y.); pinkbai@163.com (F.B.); 13834691242@163.com (J.C.); 2 Department of Pharmacology, Shanxi Medical University, 56 Xinjiannanlu, Taiyuan, Shanxi 030001, China; E-Mails: wzhaojun1025@126.com (Z.-J.W.); sxykdxyxy@163.com (X.-J.Q.); 3 Department of Physiology, Shanxi Province Key Laboratory of Cellular Physiology, Shanxi Medical University, 56 Xinjiannanlu, Taiyuan, Shanxi 030001, China

**Keywords:** epigallocatechin-3-gallate, PM_2.5_, transcription factor nuclear factor E2-related factor 2, heme oxygenase-1, human umbilical vein endothelial cells

## Abstract

Endothelial dysfunction and oxidative stress likely play roles in PM_2.5_-induced harmful effects. Epigallocatechin-3-gallate (EGCG), the major polyphenolic constituent of green tea, is a potent antioxidant that exerts protective effects on cardiovascular diseases (CVDs) in part by scavenging free radicals. The exposure to ambient fine particulate matter (PM_2.5_) is responsible for certain CVDs. The aim of the present study was to investigate whether EGCG could also inhibit PM_2.5_-induced oxidative stress by activating the nuclear factor E2-related factor 2 (Nrf2)/heme oxygenase-1 (HO-1) pathway in human umbilical vein endothelial cells (HUVECs). PM_2.5_ (200 μg/mL) increased both cell death and intracellular ROS levels significantly, whereas EGCG (50–400 μM) inhibited these effects in a concentration-dependent manner. Western blotting and PCR demonstrated that EGCG increased Nrf2 and HO-1 expression in HUVECs that had been exposed to PM_2.5_. PD98059 (a selective inhibitor of extracellular signal regulated kinase [ERK]-1/2) and SB203580 (a selective inhibitor of p38 MAPK), but not SP600125 (a selective inhibitor of c-jun N-terminal kinase [JNK]), attenuated the EGCG-induced Nrf2 and HO-1 expression. In addition, silencing Nrf2 abolished EGCG-induced Nrf2 and HO-1 upregulation and enhancement of cell viability. The present study suggests that EGCG protects HUVECs from PM_2.5_-induced oxidative stress injury by upregulating Nrf2/HO-1 via activation of the p38 MAPK and the ERK1/2 signaling pathways.

## 1. Introduction

Numerous epidemiological studies have suggested that there is a positive correlation between the exposure to ambient fine particulate matter (≤2.5 μm in aerodynamic diameter, PM_2.5_) and cardiovascular morbidity and mortality [1,2,3,4,5,6,7]. Exposure to ambient PM_2.5_ was the ninth leading factor in the global burden of disease, and contributed to 3.2 million premature deaths worldwide in 2010 [8]. There is growing concern regarding the effects of PM_2.5_ on cardiovascular diseases (CVDs). Although many studies have provided important experimental evidence to support the conclusion that PM_2.5_ is harmful to the cardiovascular system [9,10], understanding of the underlying biological mechanisms remains limited [11].

The excessive production of reactive oxygen species (ROS) is involved in the initiation and progression of CVDs [12,13] including hypertension, congestive heart failure, diabetes, atherosclerosis and ischemia. Oxidative stress is an important etiological mechanism underlying PM_2.5_-induced injury [14]. Intracellular ROS levels are regulated by antioxidant defense systems. ROS can be reduced or detoxified directly into nontoxic substances by degrading enzyme and/or be eliminated by non-enzymatic radical scavengers.

Heme oxygenase-1 (HO-1) is an inducible antioxidant enzyme that exerts cytoprotective effects in various cells [15,16,17,18,19]. The levels of HO-1 are regulated mainly by antioxidant response elements (ARE), which are expressed by the activation of nuclear factor-erythroid 2-related factor 2 (Nrf2). Normally, Nrf2 is sequestered in the cytoplasm bound to Kelch-like ECH-associated protein 1 (Keap1) [20]. The exposure to oxidants or chemoprotective compounds alters the structure of Keap1, resulting in the disaggregation of the Keap1/Nrf2 complex and the activation of Nrf2. Activated Nrf2 translocates to the nucleus where it forms a heterodimer with the Jun and Maf bZip transcription factors, which bind to the ARE [21] and induce the transcription of HO-1.

**Figure 1:**
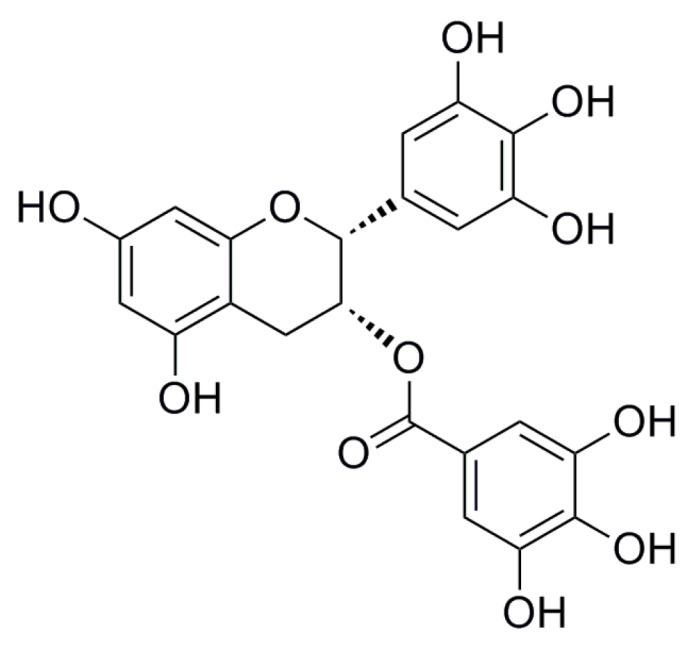
Chemical structure of epigallocatechin-3-gallate (EGCG).

Endothelial cell dysfunction is an important etiological factor in the pathogenesis and progression of CVDs. Protecting endothelial cells from oxidative injury is a strategy for the prevention and treatment of CVDs. Our previous study showed that PM_2.5_ induced oxidative stress in HUVEC. The ginsenoside Rg1 had cytoprotective effects in PM_2.5_-injured HUVECs [22]. Epigallocatechin-3-gallate (EGCG, Figure 1), the major polyphenolic constituent found in green tea, is associated with beneficial effects for the prevention of CVDs, particularly atherosclerosis and coronary heart disease. However, little is known about whether EGCG could protect against PM2.5-induced oxidative stress in human endothelial cells. The present study was designed to investigate the protective role of EGCG, and its potential mechanisms, using PM_2.5_-injured HUVECs.

## 2. Results 

### 2.1. EGCG Prevented PM_2.5_-Induced Reduction in HUVEC Viability

**Figure 2:**
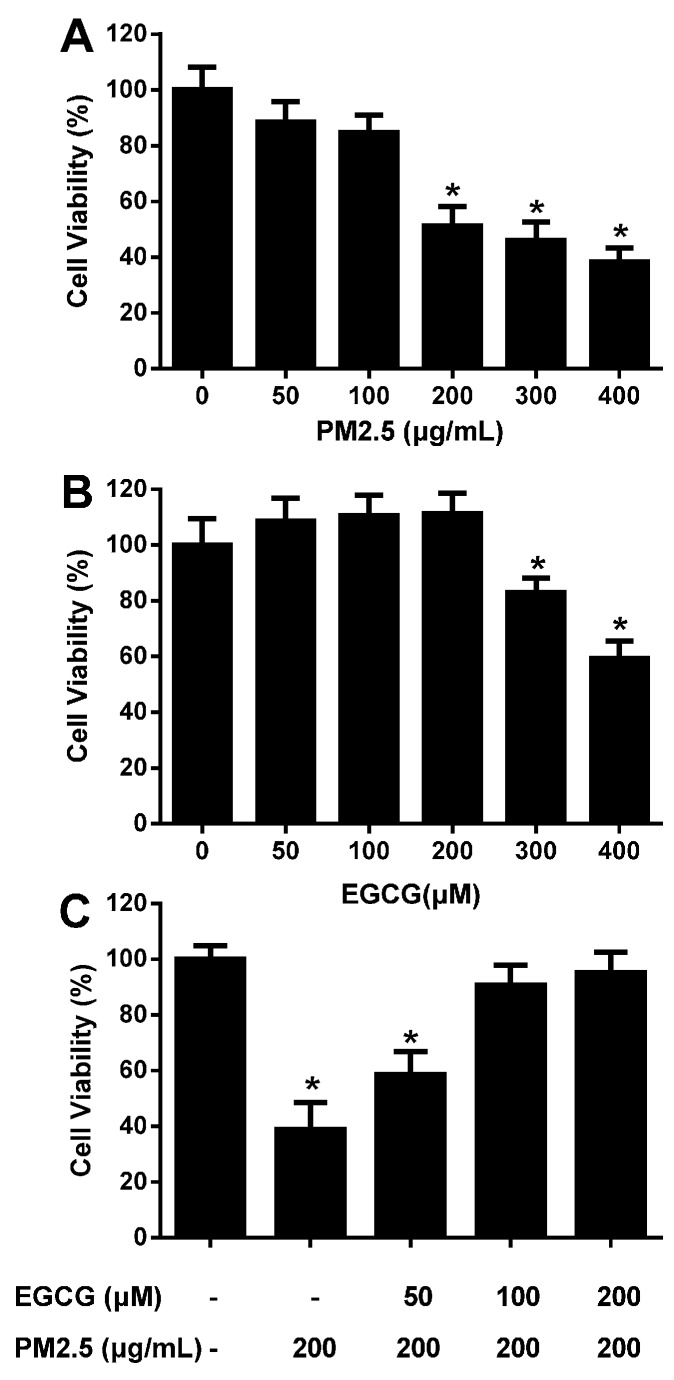
EGCG prevents PM_2.5_-induced reduction of HUVEC viability. Cell viability was evaluated using CCK-8 assays, and the viability of untreated HUVECs was taken as 100%. (**A**) HUVECs were incubated with 50–400 μg/mL PM_2.5_ for 24 h. (**B**) HUVECs were incubated with 50–400 μM EGCG for 24 h. (**C**) HUVECs were incubated with 50–200 μM EGCG for 30 min, and then co-treated with EGCG + PM2.5 (200 μg/mL) for an additional 24 h. Data are presented as means ± SEM, n = 6, * *p* < 0.05 *vs.* control (untreated HUVECs).

CCK-8 assays demonstrated that 200, 300, and 400 μg/mL PM2.5 reduced HUVEC viability to 51.24% ± 6.90%, 45.97% ± 6.64%, and 38.32% ± 4.89%, respectively; the IC_50_ value was ~200 μg/mL (Figure 2A). 

To select the optimal concentration of EGCG the concentration-dependent effects of EGCG on HUVEC viability were studied. Incubation with 50–200 μM EGCG for 24 h did not affect cell viability, whereas 300 and 400 μM reduced viability significantly (Figure 2B). Therefore, EGCG concentrations of 50–200 μM were selected for subsequent experiments. Incubating HUVECs with 50–200 μM EGCG for 24 h antagonized the effects of 200 μg/mL PM_2.5_ on cell viability in a concentration-dependent manner. EGCG (100 and 200 μM) almost abolished the PM_2.5_-induced reduction of cell viability (Figure 2C).

### 2.2. EGCG Reduced PM2.5-Induced ROS Production in HUVECs

**Figure 3:**
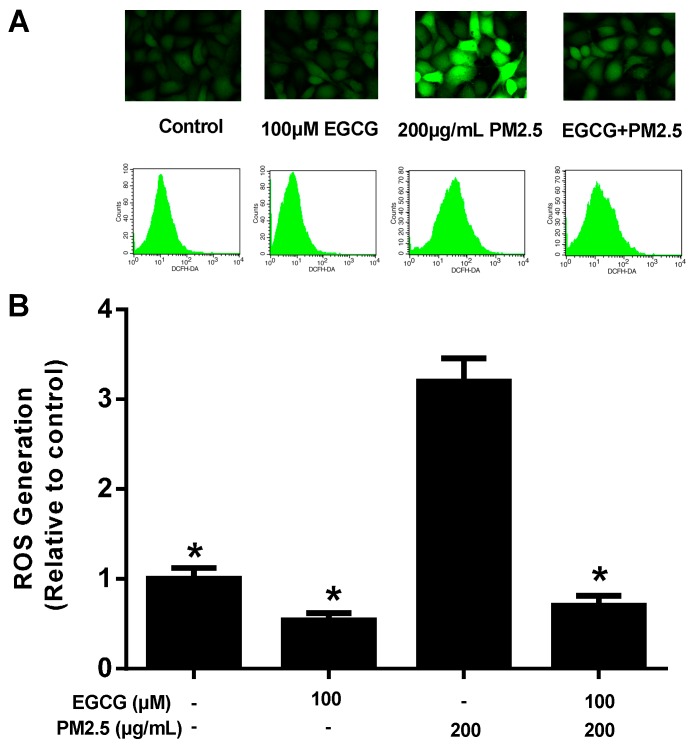
EGCG reduced PM_2.5_-induced ROS production in HUVECs. Intracellular ROS levels were determined using flow cytometry and DCFH_2_-DA. (**A**) Representative images and recordings. HUVECs were incubated without any intervention (control), EGCG alone, PM2.5 alone, or 100 μM EGCG for 30 min followed by 200 μg/mL PM_2.5_ for an additional 24 h. (**B**) Pooled data from A presented as means ± SEM, n = 6, * *p* < 0.01 *vs.* PM_2.5_-treated HUVECs.

DCFH2-DA staining showed that 200 μg/mL PM_2.5_ tripled ROS production (3.20 ± 0.08 *vs* 1.00 ± 0.12, *p* < 0.05, Figure 3). At 100 μM, EGCG alone did not affect ROS production significantly, but abolished the PM_2.5_-induced increased ROS production completely.

### 2.3. Effects of PM_2.5_ on Nrf2 and HO-1 Expression in HUVECs

**Figure 4:**
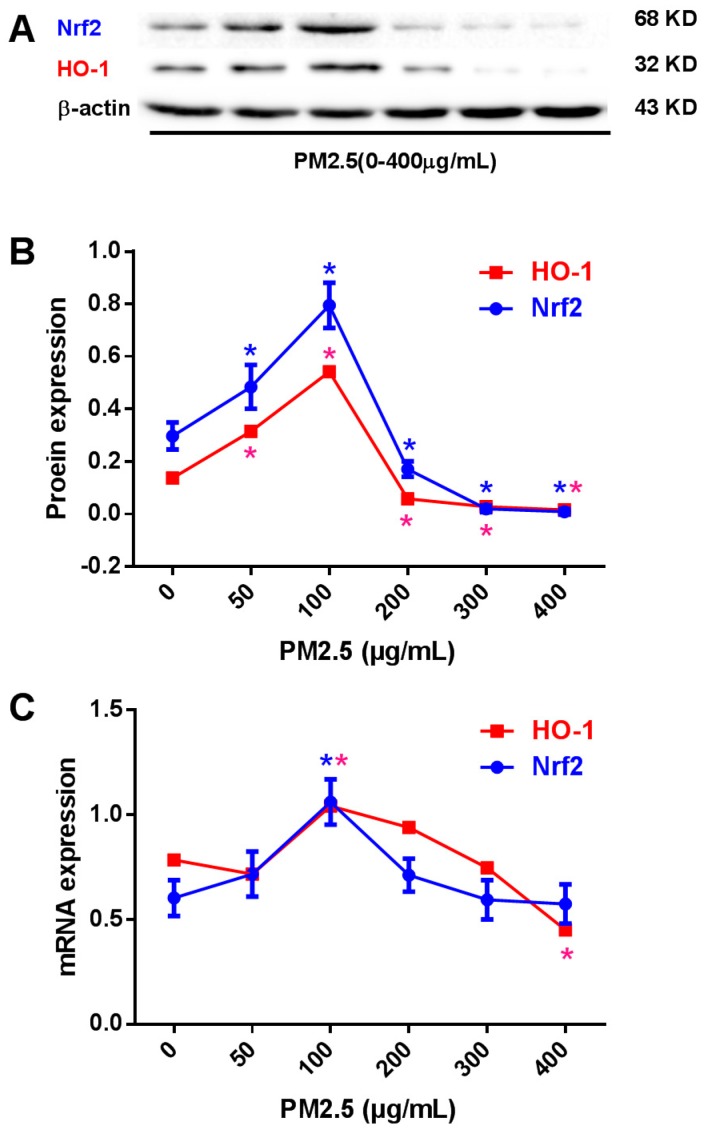
Nrf2 and HO-1 expression in PM_2.5_-treated HUVECs. (**A**) Representative images of the expression of Nrf2 and HO-1 in HUVECs treated with 0–400 μg/mL PM_2.5_ for 24 h. (**B**) Pooled data of Nrf2 and HO-1 protein expression in HUVECs. (**C**) Pooled data of *Nrf2* and *HO-1* mRNA expression in HUVECs. Data are expressed as the means ± SEM of three independent experiments. * *p* < 0.05 *vs.* HUVECs incubated without PM_2.5_.

Western blotting showed that 50 and 100 μg/mL PM_2.5_ increased Nrf2 expression to 162.6% and 267.3% and increased HO-1 expression to 230.0% and 394.9%, respectively. In contrast, at 200, 300, and 400 μg /mL, PM_2.5_ decreased Nrf2 expression to 57.6%, 6.4%, and 2.7%, and decreased HO-1 expression to 41.6%, 19.7%, and 11.7%, respectively (Figure 4A,B). Real time PCR confirmed comparable results at the mRNA level (Figure 4C).

### 2.4. EGCG Upregulated Nrf2 and HO-1 Expression in PM_2.5_-Treated HUVECs

**Figure 5:**
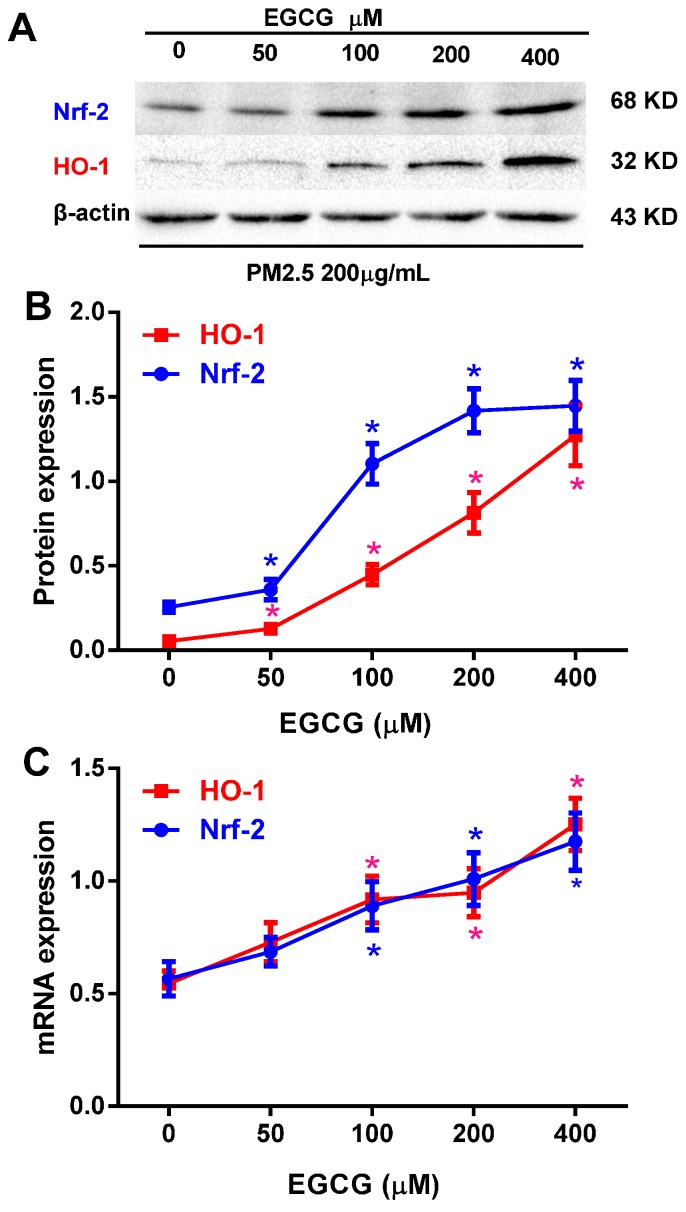
EGCG increased Nrf2 and HO-1 expression in PM_2.5_-treated HUVECs. (**A**) Representative images of Nrf2 and HO-1 protein expression in HUVECs incubated with 0–400 μM EGCG + 200 μg/mL PM_2.5_ for 24 h. EGCG was added to the medium 30 min before the addition of PM_2.5_. B and C: Pooled data of Nrf2 and HO-1 protein (**B**) and mRNA (**C**) expression in HUVECs are expressed as the means ± SEM of three independent experiments. * *p* < 0.05 *vs.* HUVECs incubated with PM_2.5_ alone.

To investigate whether EGCG could reverse the decrease in Nrf2 and HO-1 expression induced by 200 μg/mL PM_2.5_, HUVECs were incubated with 0–400 μM EGCG and 200 μg/mL PM_2.5_ for 24 h; EGCG was added to the medium 30 min before PM_2.5_. Treatment with 50–400 μM EGCG increased the expression of Nrf2 and HO-1 in HUVECs exposed to PM_2.5_ in a dose-dependent manner (Figure 5).

### 2.5. Inhibiting either ERK1/2 or P38 MAPK Abrogated the EGCG-Induced Upregulation of Nrf2 and HO-1 in HUVECs Exposed to PM_2.5_

**Figure 6:**
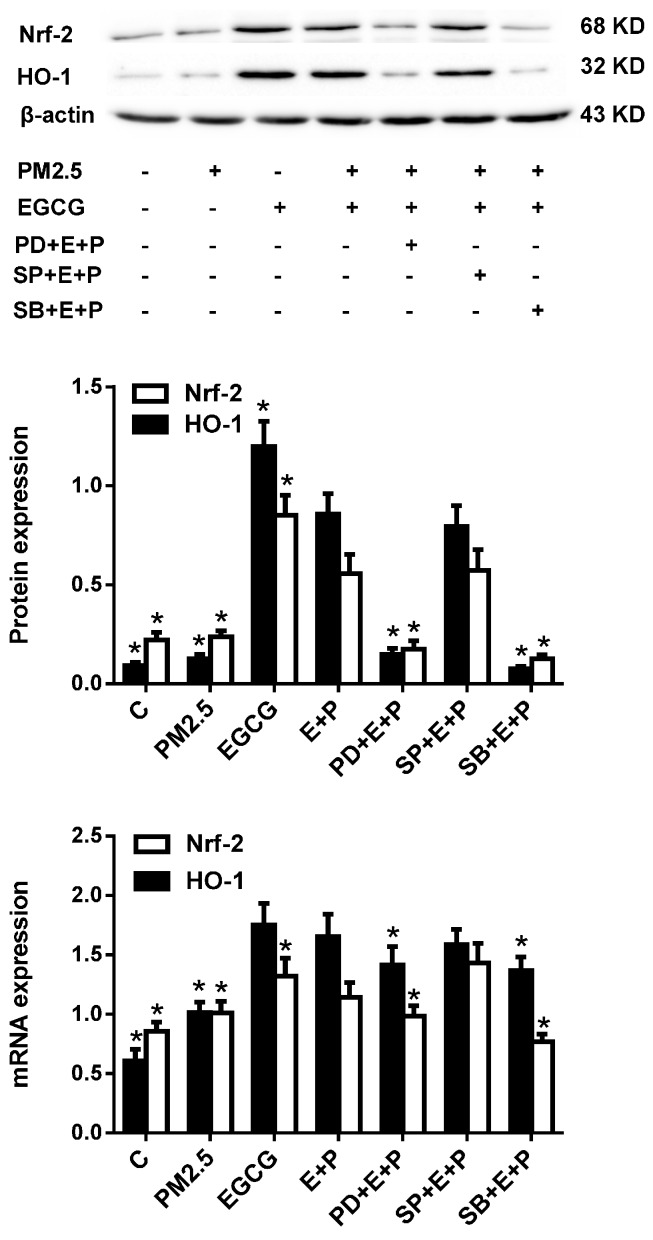
Inhibiting ERK1/2 and p38 MAPK abrogated EGCG-induced Nrf2 and HO-1 expression in PM_2.5_-treated HUVECs. Upper panel: Representative images of Nrf2 and HO-1 expression in HUVECs treated as indicated. C, cultured with medium alone for 24 h; PM_2.5_, incubated with 200 μg/mL PM_2.5_ for 24 h; EGCG, incubated with 100 μM EGCG for 24 h; E+P, incubated with 100 μM EGCG and 200 μg/mL PM_2.5_ for 24 h (EGCG was added 30 min before PM_2.5_); PD+E+P, SP+E+P, and SB+E+P, treated as described for E+P except that the ERK1/2 inhibitor PD98059, the JNK inhibitor SP600125, or the p38 MAPK inhibitor SB203580, respectively was added to the medium before EGCG. Middle panel: Pooled data of Nrf2 and HO-1 protein expression. Lower panel: Pooled data of Nrf2 and HO-1 mRNA expression. Data are expressed as the means ± SEM of three independent experiments. * *p* < 0.05 *vs.* E+P.

Because three major MAPKs (mitogen-activated protein kinases), namely extracellular signal regulated protein kinase (ERK), c-Jun N-terminal protein kinase (JNK), and p38 MAP kinase, are mediators of oxidative stress-induced endothelial injury and protection [23], we next explored the possible link between the protective effects of EGCG and these signaling pathways by assessing the effects of inhibitors of these kinases on the upregulated Nrf2 and HO-1 expression. Figure 6 reveals that pre-incubation with PD98059 (an ERK1/2 inhibitor) and SB203580 (a p38 MAPK inhibitor), but not SP600125 (a JNK inhibitor), abrogated EGCG-induced Nrf2 and HO-1 upregulation in HUVECs exposed to PM_2.5_.

### 2.6. Nrf2 Silencing Abolished the EGCG-Induced Upregulation of Nrf2 and HO-1 in HUVECs Exposed to PM_2.5_

**Figure 7:**
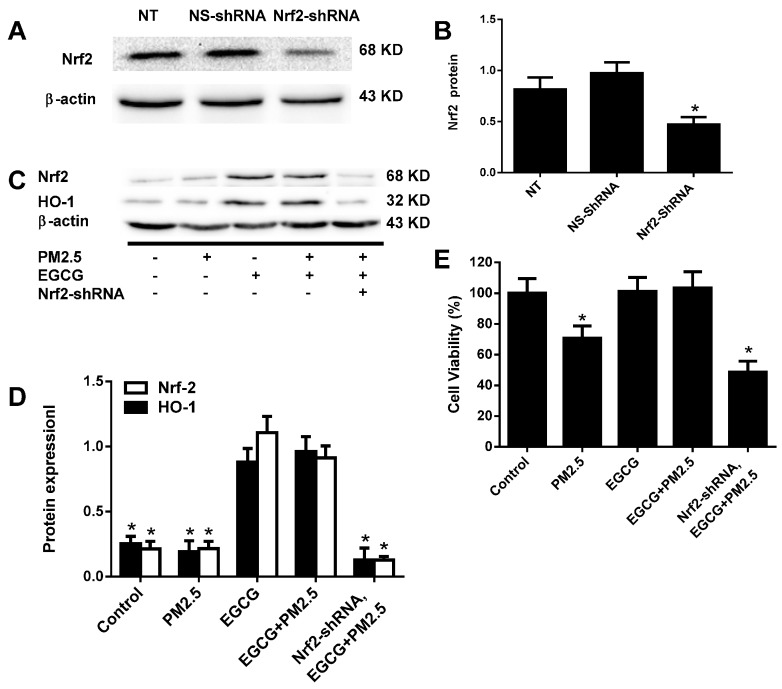
Nrf2 silencing abolished EGCG-induced Nrf2 and HO-1 upregulation in PM_2.5_-treated HUVECs. Nrf2 was silenced by transfecting HUVECs at 60%–70% confluence with Nrf2-shRNA (100 nM, Nrf2-shRNA) for 24 h. NS-shRNA, HUVECs transfected with a nonspecific shRNA (100 nM, Nrf2-shRNA-nonsilencing control); NT, non-transfected control HUVECs. (**A**) Western blotting demonstrating the efficiency of Nrf2 silencing using β-actin as a loading control. (**B**) Pooled data from A are expressed as the means ± SEM of three independent experiments. * *p* < 0.05 *vs.* NS-shRNA. (**C**) Representative images of Nrf2 and HO-1 protein expression in HUVECs treated as indicated. (**D**) Pooled data from C are expressed as the means ± SEM of three independent experiments. * *p* < 0.05 *vs.* EGCG+PM_2.5_. (**E**) Cell viabilities in HUVECs treated as indicated are expressed as the means ± SEM of six independent experiments. * *p* < 0.05 *vs.* EGCG+PM_2.5_.

A *Nrf2* silencing study demonstrated that *Nrf2* expression plays an important role in the EGCG-induced upregulated expression of Nrf2 and HO-1 and cell viability (Figure 7). 

## 3. Discussion

The most important findings of the current study can be summarized as follows. (1) Water-soluble extracts of PM_2.5_ increased ROS production and decreased viability in HUVECs. (2) EGCG reversed the effects of PM_2.5_ in a dose-dependent manner. (3) EGCG protected HUVECs from PM_2.5_-induced injury by increasing the expression of Nrf2/HO-1 via the ERK1/2 and p38 MAPK pathways.

Exposure to PM_2.5_ was recently identified as an important cardiovascular risk factor, but whether it impairs endothelial function through production of reactive oxygen species (ROS) in endothelial cells is not well known. Nonetheless, antioxidant therapies have been gaining recognition as strategies to reduce ROS in the vasculature and thereby diminish their detrimental effects [24]. The production of ROS has been argued to play an important role in the primary cytotoxic effects of PM_2.5_ [25]. In this study, a human umbilical vein endothelial cell line was used as an *in vitro* model to measure cell viability and intracellular ROS upon PM2.5 exposure. We observed that high-doses of PM_2.5_ increased intracellular ROS and decreased cell viability, which was consistent with our previous study [22]. In the present study, MAPK inhibitor and Nrf2 silencing was used to study the protective role of EGCG against PM_2.5_-induced HUVEC injury.

One of the possible mechanisms by which EGCG prevents PM_2.5_-induced HUVEC injury might be the activation of the Nrf2/HO-1 pathway. Pullikotil *et al*. found that EGCG increased expression of Nrf2 to drive the expression of HO-1 and thereby increase HO-1 activity in human aortic endothelial cells [26]. Wu *et al.* demonstrated that EGCG induces HO-1 expression in a concentration- and time-dependent manner in bovine aortic endothelial cells [27]. It has been shown that pretreating endothelial cells with EGCG exerted significant cytoprotective effects against H_2_O_2_, suggesting that the induction of HO-1 is an important component of the protective effects against oxidative stress [28]. We demonstrated that EGCG exerted powerful antioxidant effects and reversed PM_2.5_-induced oxidative stress in HUVECs. The present results showed that EGCG can decrease intercellular ROS production, increase Nrf2/HO-1 expression and cell viability in the setting of PM_2.5_ exposure. These results support that EGCG protects HUVEC from high dose PM_2.5_-induced injury at least in part through Nrf2/HO-1 activation.

An extensive range of experimental and epidemiological studies have suggested that airborne particles, particularly PM_2.5_, play an important role in the overall development of CVDs [29,30,31]. Physiologically, the generation and elimination of ROS is balanced by free radical scavenging and the upregulation of antioxidant enzymes. Nrf2 is a key regulatory transcription factor that induces antioxidant and detoxification genes that protect against the adverse effects of exogenous or endogenous oxidative stress. Normally, Nrf2 is bound to Keap1 and inactivated in the cytoplasm. EGCG can dissociate Nrf2 from Keap1 to activate it. Activated Nrf2 then enters the nucleus and binds to AREs to stimulate the expression of target genes such as HO-1. HO-1 detoxifies ROS [32]. 

The MAPK family is involved in the anti-oxidant, anti-inflammatory, anti-proliferative, and anti-thrombotic effects of EGCG [26,33]. In the present study, both SB203580 and PD98059, inhibitors of p38 MAPK and ERK1/2, respectively, decreased intracellular ROS by downregulating Nrf2/HO-1 in HUVECs and abolishing the protective effects of EGCG. Therefore, the activation of p38 MAPK and ERK1/2 might be required for the protective effects of EGCG in HUVECs. 

It was reported that Nrf2−/− (knockout) mice are highly sensitive to cytotoxic electrophiles compared with Nrf2+/+ (wild-type) mice because of decreased levels of phase II detoxification enzymes and antioxidant proteins [34]. In the present study, we demonstrated that Nrf2 silencing abolished the EGCG-induced upregulation of cell viability and Nrf2 and HO-1 in HUVECs exposed to PM2.5, supporting that Nrf2 plays a key role in EGCG-induced upregulated expression of HO-1.

In conclusion, the present study provides evidence that EGCG protects HUVECs from oxidative stress induced by high-dose PM2.5 through activation of the p38 MAPK and ERK1/2 pathways, which in turn increase the expression of Nrf2/HO-1. 

## 4. Experimental Section

### 4.1. Reagents and Cell Culture 

Primary antibodies against HO-1 and Nrf2 were purchased from Abcam, (Cambridge, UK). The MAPK pathway inhibitors were provided by Santa Cruz Biotechnology (Santa Cruz, CA, USA). The human specific Nrf2 shRNA and scrambled control duplexes were provided by GeneCopoesia (Rockville, MD, USA). Dulbecco’s modified Eagle’s medium (DMEM) and fetal bovine serum (FBS) were purchased from Gibco BRL (New York, NY, USA). EGCG (>98% purity) was purchased from Solarbio Co., Ltd (Peking, China). Enhanced chemiluminescence (ECL) reagent was obtained from Beyotime (Jiangsu, China). DAPI was obtained from Sigma (St. Louis, MO, USA). Lipofectamine 2000 reagent, dimethyl sulfoxide (DMSO), and penicillin-streptomycin were purchased from Invitrogen (Carlsbad, CA, USA). HUVECs were purchased from Shanghai Institute of Cell Biology, Chinese Academy of Sciences, and were grown in DMEM supplemented with 10% (*v*/*v*) heat-inactivated fetal bovine serum and 1% (*v*/*v*) penicillin–streptomycin at 37 °C in a humidified atmosphere containing 5% CO_2_.

### 4.2. Ambient PM2.5 Water-Soluble Extracts

Fifty milligrams of PM2.5 (SRM#1650b, NIST, Boulder, CO, USA) was suspended in 5 mL of phosphate buffered saline (PBS) for 24 h and sonicated for 20 min. The PM2.5 suspension was then centrifuged at 20,000 rpm for 10 min at 4 °C, and any impurities were removed using a 0.22-μm syringe filter.

### 4.3. Cytotoxicity Assays

Cell viability was measured using Cell Counting Assay Kit 8 (CCK-8, Dojindo, kumamoto, Japan) according to the manufacturer’s protocol. HUVECs (5 × 10^3^ cells/well) were seeded into 96-well plates in culture medium, maintained in regular growth medium overnight, and then treated with EGCG and PM2.5 for 24 h. EGCG was added to the medium 30 min before the addition of PM2.5. Ten-microliters of CCK-8 solution were added to each well for 1 h, and the absorbance at 450 nm was measured using a microplate reader (Molecular Devices, Sunnyvale, CA, USA).

### 4.4. Determining Intracellular ROS Production

Intracellular ROS production was measured using flow cytometry and light microscopy. After treatment the cells were washed and incubated with DCFH_2_-DA (10 μmol/L; Sigma) for 20 min at 37 °C in the dark. The fluorescence corresponding to intracellular ROS levels was monitored at 488 nm (excitation) and 519 nm (emission) using a confocal fluorescence microscope (Olympus FV1000, Tokyo, Japan) and analyzed using flow cytometry.

### 4.5. Western Blotting

HUVECs were treated as described above, washed in PBS, lysed, and harvested. Following centrifugation at 14,000 rpm for 15 min at 4 °C, the supernatants were collected and stored at −70 °C until use. The protein concentrations were determined using BCA protein assay kits (Thermo Scientific, Hudson, NH, USA). After the addition of sample loading buffer, proteins were resolved using 12% SDS-polyacrylamide gel electrophoresis. The proteins were transferred to polyvinylidene difluoride membranes (Millipore, Bedford, MA, USA) at 15 eV for 15 min, and blocked in 5% dry milk in PBST (PBS containing 0.1% Tween-20e for 2 h. The blots were then incubated with primary antibodies diluted in PBST overnight at 4 °C, washed three times with PBST, and incubated with horseradish peroxidase-conjugated secondary antibodies in PBST for 1 h. The blots were washed again three times with PBST, and the resulting immunoreactive protein complexes were detected using ECL detection reagent according to the manufacturer’s instructions.

### 4.6. Real-Time PCR

Total RNA was isolated from treated HUVECs using RNeasy spin columns (Qiagen, Hilden, Germany). Real-time PCR was performed in triplicate on a Stratagene Mx3005p multiplex quantitative PCR system (Stratagene, La Jolla, CA, USA) using 100 ng RNA, 12.5 μL 2× QuantiFast SYBR (Qiagen), and 1 μM each of the forward and reverse primers in a final reaction volume of 25 μL. Samples were incubated at 95 °C for 5 min, followed by 40 cycles of denaturation at 95 °C for 10 s and annealing and extension at 60 °C for 30 s. All amplifications were normalized to β-actin, and the amount of the target gene was then presented as 2^−ΔΔCt^ to determine the expression of *Nrf2* and *HO-1* mRNA. The primers used for real-time PCR were as follows: Nrf2 forward, 5'-GAATTGCCTGTAAGTCCTGGTC-3', and reverse 5'-GGTGAAGGCTTTTGTCATTTTC-3'; HO-1 forward 5'-CTTCTTCACCTTCCCCAACA-3' and reverse 5'-ATTGCCTGGATGTGCTTT TC-3'; and β-actin forward 5'-GGAAATCGTGCGTGACATTA-3' and reverse 5'-GGAGCAATGAT CTTGATCTTC-3'.

### 4.7. Nrf2-shRNA Transient Transfection

HUVECs were transfected with shRNA specifically targeting human *Nrf2* (#HSH011800-5-CH1, sequence (5' to 3', CCGACAACCACTACCTGA) using Lipofectamine 2000 reagent according to the manufacturer’s instructions. Cells were used in experiments 48 h after transfection (to allow the turnover of residual Nrf2 protein and block *de novo* synthesis of Nrf2). The successful knockdown of the target proteins was confirmed using western blotting.

### 4.8. Statistical Analysis

Statistical analysis was performed using SPSS 13.0 (SPSS Inc., Chicago, IL, USA). One-way ANOVA was used to compare different treatment regimens. All results are expressed as means ± standard error of the mean (SEM). *p* < 0.05 was considered to be statistically significant.
